# Total knee arthroplasty: Limb length discrepancy and functional outcome

**DOI:** 10.4103/0019-5413.65159

**Published:** 2010

**Authors:** Shrinand V Vaidya, Mihir R Patel, Atul N Panghate, Parthiv A Rathod

**Affiliations:** 1 Department of Orthopedic Surgery, King Edward VII Memorial Hospital and Seth Gordhandas Sundardas Medical College, Mumbai, India; 2 Cumballa Hill Hospital Superspeciality Center for Computer-Assisted Joint Replacement Surgery, Gowalia Tank, Kemp’s Corner, Mumbai, India

**Keywords:** Limb length discrepancy, osteoarthritis knee, total knee arthroplasty

## Abstract

**Background:**

Limb length discrepancy and its effects on patient function have been discussed in depth in the literature with respect to hip arthroplasty but there are few studies that have examined the effect on function of limb length discrepency following total knee arthroplasty (TKA). The aim of this study was to determine whether limb length discrepancy after TKA in patients with bilateral osteoarthritis of knee with varus deformity affects functional outcome.

**Materials and Methods:**

Fifty-four patients with bilateral osteoarthritis of knee with varus deformity, who were operated for total knee arthroplasty from 1996 to 2008, were reviewed retrospectively. The patients were divided into two groups. Thirty patients (mean age 64 years) were operated for unilateral TKA and thirty patients (mean age 65.8 years) were operated for bilateral total knee arthroplasty. Six patients underwent staged surgery and were included in both groups as the time interval between the two surgeries was more than the minimum 6-month follow-up period specified for inclusion in the study. The limb length discrepancy was measured and statistically correlated with the functional component of the Knee Society Score.

**Result:**

In the unilateral group (n=30), the mean limb length discrepancy was 1.53 cm (range: 0-3 cm) and the mean functional score was 73 (range: 45-100). In the bilateral group (n=30), the mean limb length discrepancy was 0.5 cm (range: 0-2 cm) and the mean functional score was 80.67 (range: 0-100). A statistically significant negative correlation was found between limb length discrepancy and functional score in the unilateral group (Spearman correlation coefficient, r =−0.52, *P*=0.006), while no statistically significant correlation was found in the bilateral group (Spearman correlation coefficient, r = −0.141, *P*=0.458).

**Conclusion:**

Limb length discrepancy affects functional outcome after total knee arthroplasty, especially so in patients of bilateral osteoarthritis with varus deformity undergoing surgery of only one knee.

## INTRODUCTION

Improved surgical techniques and rehabilitation protocols have resulted in excellent knee function and range of motion following total knee arthroplasty. Nevertheless, there remain 15-20% of patients with persistent dysfunction that is difficult to treat.^1^–^3^ Although problems after total knee arthroplasty are frequently linked to prosthetic malalignment, radiographic loosening, and comorbidities, some cases are related to functional problems that are less evident clinically and/or radiographically.

Ulrich *et al*.^4^ identified six functional abnormalities: knee flexion contracture, quadriceps muscle weakness, knee flexion deficit, limb length difference, foot and ankle malalignment, and peroneal nerve entrapment. Patients having these problems showed only minimal improvement in terms of both function and pain relief after the initial physical rehabilitation program. With customized rehabilitation and physical therapy modalities, there was improvement in the clinical outcome in the above group of patients.

Functional problems following total knee arthroplasty may be incapacitating as a result of persistent pain,^5^ instability,^6^ and limited range of motion^7^,^8^ Patients who experienced more pain and functional impairment after total knee arthroplasty were less likely to be satisfied with the procedure.^9^

The aim of this study was to determine whether limb length discrepancy after TKA in patients with bilateral osteoarthritis of knee with varus deformity affects functional outcome.The aim of this study was to investigate for the presence of limb length discrepancy after total knee arthroplasty, the amount of discrepancy, patient perception of the limb length discrepancy, its effects on the patient function, and any difference between patients undergoing unilateral and bilateral total knee arthroplasty with regard to function due to limb length discrepancy.

## MATERIALS AND METHODS

This was a retrospective study conducted in a tertiary level hospital by a specialist arthroplasty unit. Fifty-four patients of bilateral osteoarthritis of the knee with varus deformity who were operated for unilateral or bilateral total knee arthroplasty consecutively and had minimum 6 months follow up were enrolled into this study. The surgeries were performed over the time period from 1996 to 2008. Cases of osteoarthritis of the knee with valgus deformity and of rheumatoid arthritis were excluded from the study.

Thirteen patients were males and 41 were females. The mean age of patient was of 64.9 years (range 48 to 83 years). The 54 patients were divided into two groups. Group A (30 patients) were operated for unilateral total knee arthroplasty and Group B (30 patients) for bilateral total knee arthroplasty. Six patients were included in both the unilateral and the bilateral group; these six patients underwent staged surgeries, during the time interval of this study, with a gap more than 6 months, hence were available for evaluation in both groups. They were thus included in the unilateral group after being operated for one knee and then in the bilateral group following the second knee arthroplasty. The 24 patients who underwent unilateral total knee arthroplasty did not get operated on the second knee for reasons that were personal or financial.

All the surgeries were performed by the same arthroplasty surgeon using the midvastus approach^10^,^11^ under thigh tourniquet. Tibial preparation was followed by femoral and patellar preparation. The patella was replaced in all the cases. All the components were cemented. Eighty-four knees were operated in all; in 56 cases the Press-Fit Condylar (PFC) sigma rotating-platform high-flexion (RP-F) knee implant (DePuy International) was used, in 26 cases the Press-Fit Condylar (PFC) Sigma Rotating-Platform (RP) knee implant (DePuy International), and in 2 cases the Insall-Burstein II (IB-II, Zimmer) knee implant. All but two of the bilaterally operated patients had similar prosthesis implanted in both the knees. Among the two bilaterally operated patients who had different prosthesis in the two knees, the first patient had IB-II in the right and PFC-RP in the left knee, while the second patient had PFC-RP in the right knee and PFC-RP-F in the left knee. An indwelling epidural catheter was placed for 48 hours postoperatively for pain relief. Postoperatively, a compressive dressing and closed suction drain was applied. which was removed at 48 hours, Postoperative rehabilitation consisted of ankle pump and active knee range-of-motion exercises from the day of surgery, under the supervision of a physiotherapist. Gait training and full weight bearing was encouraged from day 2, initially with a walker and then with a tripod walking stick as per patient comfort and confidence. Staircase climbing was begun on day 5. The patient was discharged on the sixth postoperative day. Further rehabilitation was carried out by a home visiting physiotherapist. It was a specifically prescribed regimen consisting of global isometric exercises, resistive exercises, walking with stick and then without depending on lurch. Outdoor walking was started by the third week.

The unilateral TKA group consisted of 30 patients, with 7 males and 23 females. The mean age in this group was 64 years (range 48 to 80 years) (mean of 64 years). The bilateral TKA group consisted of 30 patients, with 6 males and 24 females. The mean age was 65.8 years (range 54-83 years). Of the 30 patients who had bilateral total knee arthroplasty, 10 were operated for both the knees simultaneously, while the remaining 20 underwent staged procedures, with the time interval between the two surgeries ranging from 1 week to 12 years. The time interval between the staged surgeries was less than six months for 13 patients and more than six months for the remaining 7 patients. The evaluation was done at recent follow up for all the cases.

Limb length measurement was done in the supine position. The pelvis was squared, i.e., the line joining the anterior superior iliac spines was kept perpendicular to the long axis of the body (xiphisternum to pubic symphysis). The lower limbs were placed parallel to the long axis of the body, and limb length measurement (in centimeters) was done from the anterior superior iliac spine to the medial malleolus using a measuring tape. The measurement was taken twice by two different observers and the mean of the two values was recorded as the limb length.^12^ We used the supine position to measure limb length because it is possible to square the pelvis and hence eliminate any suprapelvic cause of limb length discrepancy. The measurement of limb length discrepancy in the standing position and the use of blocks was not done as the patients were not comfortable standing in this position because of pain from the arthritic knee. This was especially true for the eight patients above 70 years of age in the unilateral group. Similarly a standing scanogram would also require the patient to stand erect with the patellae facing forward, while three radiographs are taken, centred on the hip, knee and ankle and a scanogram would expose each patient to three radiation exposures.

An anteroposterior (AP) roentgenogram of the operated limb was obtained in the supine position with the patella facing the ceiling. Similarly, an AP standing roentgenogram of the operated limb was obtained in the standing position with the patella facing forward. The x-ray beam was directed at the joint line. A lateral view was obtained in the supine position, with the beam directed perpendicular to that in the AP view. A skyline view was obtained to study the patellar component and tracking. Alignment, component positioning, and loosening were evaluated from the above roentgenograms by an independent observer as per the Knee Society total knee arthroplasty roentgenographic evaluation and scoring system.^13^ The knee alignment was measured from the AP standing roentgenogram by drawing the mid-medullary lines of the femur and tibia and mearsuring the angle at the intersection. The mean knee valgus was 5.37xxd (range 5° to 7°). The femoral component positioning was measured from the lateral roentgenogram and ranged from 3° flexion to 3° extension. The tibial component positioning was measured from the AP supine and lateral roentgenograms and ranged from 3° varus to 3° valgus and 3° flexion to 3° extension. Loosening was assessed for the femoral component on the lateral roentgenogram in the seven zones.^13^ Similarly, loosening for the tibial component was assessed on the AP supine and lateral roentgenograms in seven and three zones, respectively. For patellar component loosening, the skyline view was studied, and loosening was noted in the five zones. Patellar tracking was evaluated clinically as well as from the skyline view by observing the relationship between the patella and the femur. Stability was measured clinically in the AP and mediolateral plane by an independent observer and scoring was done according to the Knee Society Clinical Rating System (Insall Modification-1993).^14^ Range of motion, flexion deformity, and extensor lag were measured clinically using a long-arm goniometer. The scoring was done according to the Knee Society Clinical Rating System. Range of motion was recorded and scored as 1 point for every 8° of range. Pain score was recorded and tabulated, according to the Knee Society Clinical Rating System, as pain on walking, pain on staircase climbing, and pain at rest. Rest pain score was deducted from the total pain score on walking and stair climbing and recorded as pain score. The functional score takes into account walking distance, stair climbing, and use of walking aids. The score was recorded as per the Knee Society Clinical Rating System and tabulated [Table 1].

**Table 1:** The Knee Society Clinical Rating System (Insall Modification - 1993)

	Knee score	
Finding	Description	Score
Pain		50 (maximum)
Walking	None	35
	Mild or occasional	30
	Moderate	15
	Severe	0
Stairs	None	15
	Mild or occasional	10
	Moderate	5
	Severe	0
Range of motion	8 degree = 1point	25 (maximum)
Stability		25 (maximum)
Medial/Lateral	0 – 5 mm	15
	5 -10 mm	10
	> 10 mm	5
Anterior/Posterior	0 – 5	10
	5 – 10	8
	> 10	5
Deductions		
Extensor lag	None	0
	< 4 degrees	-2
	5 – 10 degrees	-5
	> 11 degrees	-10
Flexion contracture	< 5 degrees	0
	6 – 10 degrees	-3
	11 – 20 degrees	-5
	> 20 degrees	-10
Malalignment	5 – 10 degrees	0
	(5 degrees)	(- 2 )
Pain at rest	Mild	-5
	Moderate	-10
	Severe	-15
	Symptomatic plus objective	0
Knee score	100 (maximum)	
Functional score		
Finding	Description	Score
Walking	Unlimited	50
	> 10 blocks	40
	5 – 10 blocks	30
	< 5 blocks	10
	Housebound	0
Stairs	Normal up and down	50
	Normal up and down with rail	40
	Up and down with rail	30
	Up with rail; unable down	15
	Unable	0
Functional deductions	Cane	- 5
	Two canes	- 10
	Crutches or walker	- 20
Functional score	100 (Maximum)	
		

Only the functional component of the Knee Society Clinical Rating System^14^ was used, as the aim of the study was to find out if there was any correlation between limb length discrepancy and patient function after total knee arthroplasty. This scoring was done by the same observer for all the patients. The maximum functional score was ‘100’ and the minimum was ‘0.’ Any negative score was recorded as ‘0.’ Each patient was asked if he or she perceived any limb length discrepancy and the answer was recorded as ‘Yes’ or ‘No.’

## RESULTS

In the Group A (unilateral group), mean limb length discrepancy (ie the operated limb gained length compared to the unoperated contralateral limb) was 1.53 cm (range 0 to 3 cm). Five patients (16.67%) had no limb length discrepancy, five (16.67%) had limb length discrepancy of 1 cm, nineteen (63.33%) had limb length discrepancy of 2 cm, and one (3.33%) had a limb length discrepancy of 3 cm.

**Figure 1:**
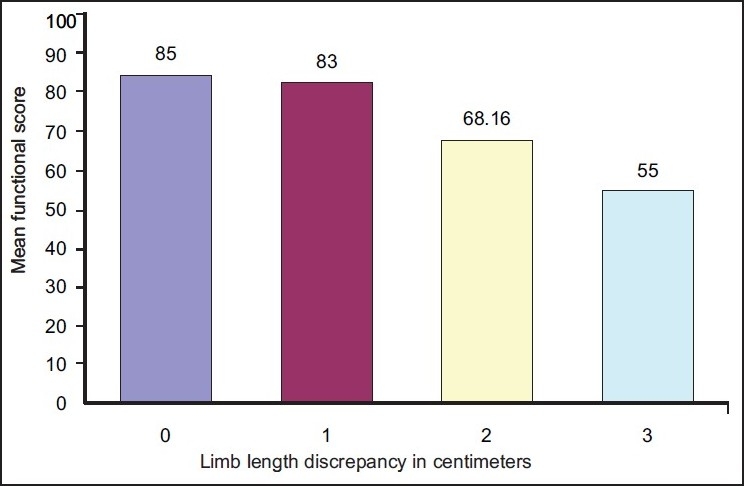
Bar diagram showing limb length discrepancy and mean functional score for the unilateral group

The mean functional score in this group, was 73 (95% CI: 66.73 to 79.27) with range from 45 to 100. The standard deviation was 16.79. The mean functional score of patients with no limb length discrepancy was 85. Those with a limb length discrepancy of 1 cm had a mean functional score of 83, those with limb length discrepancy of 2 cm had a mean score of 68.16, and those with limb length discrepancy of 3 cm had a mean functional score of 55 [Figure 1]. A statistically significant negative correlation was found between limb length discrepancy and the functional score for the unilateral group (Spearman correlation coefficient r=−0.52, *P*=.006).

A total of eight patients (26.7%) perceived the limb length discrepancy, while the remaining 22 patients (73.3%) did not. Of the eight patients who perceived limb length discrepancy, seven (87.5%) had limb length discrepancy of 2 cm and the remaining one (12.5%) patient had a limb length discrepancy of 3 cm. None of the patients with limb length discrepancy of 1 cm perceived it. Of the 19 patients with limb length discrepancy of 2 cm, seven (36.8%) perceived the limb length discrepancy. The single patient with a 3 cm limb length discrepancy also perceived the same [Tables 2 and 3].

**Table 2:** Data chart for the unilateral group

Patient	Right (cm)	Left (cm)	L.L.D. (cm)	R.O.M. (degrees)	Perception
1	78	76	2	130	No
2	77	75	2	130	No
3	80	78	2	120	No
4	87	85	2	120	No
5	87	85	2	120	Yes
6	78	75	3	100	Yes
7	75	77	2	110	No
8	82	82	0	120	No
9	85	87	2	130	No
10	73	72	1	120	No
11	75	75	2	100	No
12	72	72	0	100	No
13	73	72	1	120	No
14	72	70	2	130	Yes
15	74	74	0	100	No
16	72	70	2	100	Yes
17	78	80	2	100	No
18	76	76	0	110	No
19	73	75	2	110	Yes
20	77	78	1	120	No
21	75	73	2	120	Yes
22	75	77	2	110	No
23	72	72	0	120	No
24	71	72	1	120	No
25	81	80	1	110	No
26	81	83	2	100	Yes
27	74	72	2	100	No
28	71	69	2	110	Yes
29	73	71	2	110	No
30	74	72	2	110	No

**Figure 2:**
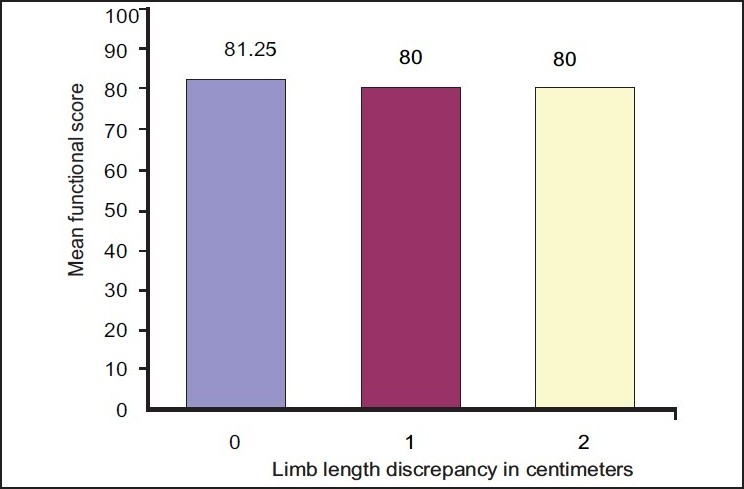
Bar diagram showing limb length discrepancy and mean functional score for the bilateral group

In the group B (bilateral group) mean limb length discrepancy was 0.5 cm (range 0 cm to 2 cm). Sixteen patients (53.33%) had no limb length discrepancy, thirteen (43.33%) had limb length discrepancy of 1 cm, and 1 (3.33%) had a limb length discrepancy of 2 cm. In the bilateral group the functional score ranged from 0 to 100, with a mean of 80.67 (95% CI: 72.75 to 88.58); the standard deviation was 21.2. The mean functional score of patients with no limb length discrepancy was 81.25, that of those with 1-cm limb length discrepancy was 80.00, and that of those with 2 cm limb length discrepancy was 80.00 [Figure 2]. No statistically significant correlation was found between limb length discrepancy and functional score in the bilateral group (Spearman correlation coefficient r= −0.141, *P*=.458). None of the patients in the bilateral group perceived any limb length discrepancy.

Six patients in the study were included in both the groups. After unilateral total knee arthroplasty, five patients had limb length discrepancy of 2 cm and one had limb length discrepancy of 3 cm. Two patients perceived the limb length discrepancy. After bilateral total knee arthroplasty, only one patient had limb length discrepancy of 1 cm; the remaining five patients had no limb length discrepancy. The one patient with limb length discrepancy did not perceive it. The mean functional score after unilateral total knee arthroplasty was 64.17 and that after bilateral total knee arthroplasty was 80.00. The difference in functional score was statistically significant (paired sample *t* test, *P*=.005 at 95% confidence interval) [Tables 4 and 5].

**Table 3:** Scoring sheet for unilateral group

Patient	Pain walk	Pain stair	Rest pain	Pain score	R.O.M. score	Knee score	Walking	Stairs	Deduction	Functional score
1	30	5	0	35	16.25	76.25	30	30	0	60
2	30	10	0	40	16.25	81.25	40	30	-5	65
3	15	10	0	25	15	65	40	30	-5	65
4	35	10	0	45	15	85	40	30	-5	65
5	30	5	0	35	15	75	40	40	-5	75
6	15	5	-5	15	12.5	52.5	30	30	5	55
7	35	10	0	45	13.75	83.75	50	50	0	100
8	15	15	0	30	15	70	50	50	0	100
9	30	10	0	40	16.25	81.25	40	50	0	90
10	30	5	0	35	15	75	30	30	0	60
11	15	5	-5	15	12.5	52.5	30	15	0	45
12	15	10	0	25	12.5	62.5	40	40	0	80
13	35	10	0	45	15	85	40	40	0	80
14	15	15	0	30	16.25	71.25	50	30	-5	75
15	30	10	0	40	12.5	77.5	40	30	-5	65
16	35	10	0	45	12.5	82.5	40	50	0	90
17	30	10	0	40	12.5	77.5	40	30	-5	65
18	30	10	0	40	13.75	78.75	50	50	0	100
19	30	10	0	40	13.75	78.75	30	30	-5	55
20	30	10	0	40	15	80	40	40	0	80
21	30	15	0	45	15	85	30	30	-5	55
22	35	15	0	50	13.75	88.75	50	50	-5	95
23	30	10	0	40	15	80	50	30	0	80
24	30	10	0	40	15	80	50	50	0	100
25	35	15	0	50	13.75	88.75	50	50	-5	95
26	30	10	0	40	12.5	77.5	30	30	-5	55
27	15	15	0	30	12.5	67.5	30	30	-5	55
28	35	15	0	50	13.75	88.75	40	30	-5	65
29	30	10	0	40	13.75	78.75	30	30	-5	55
30	30	10	0	40	13.75	78.75	40	30	-5	65

**Table 4:** Data chart for the bilateral group

Patient	Right (cm)	Left (cm)	L.L.D. (cm)	R.O.M. right (degrees)	R.O.M. left (degrees)	Perception
1	78	78	0	130	120	No
2	77	78	1	130	120	No
3	80	80	0	120	120	No
4	87	87	0	90	100	No
5	87	87	0	120	120	No
6	78	78	0	100	100	No
7	75	75	0	130	110	No
8	75	74	1	100	100	No
9	95	96	1	110	110	No
10	78	78	0	120	120	No
11	78	80	2	100	110	No
12	85	85	0	120	120	No
13	79	80	1	120	120	No
14	80	80	0	110	120	No
15	73	74	1	110	110	No
16	78	79	1	120	100	No
17	74	74	0	100	100	No
18	76	77	1	120	120	No
19	87	87	0	120	120	No
20	78	78	0	120	120	No
21	76	76	0	120	120	No
22	71	70	1	120	110	No
23	70	71	1	120	120	No
24	80	81	1	130	120	No
25	70	71	1	90	100	No
26	75	76	1	100	110	No
27	74	74	0	100	110	No
28	78	78	0	120	90	No
29	75	75	0	90	100	No
30	70	71	1	90	100	No

**Table 5:** Scoring sheet for bilateral group

Patient	Pain walk	Pain stair	Rest pain	Pain score	R.O.M. score right	R.O.M. score left	Knee score right	Knee score left	Walking	Stairs	Deductions	Functional score
1	30	10	0	40	16.25	15	81.25	80	40	40	0	80
2	35	10	0	45	16.25	15	86.25	85	40	40	-5	75
3	35	15	0	50	15	15	90	90	50	50	-5	95
4	35	15	0	50	11.25	12.5	86.25	87.5	40	40	-5	75
5	30	10	0	40	15	15	80	80	50	40	0	90
6	30	10	0	40	12.5	12.5	77.5	77.5	40	30	-5	65
7	35	15	0	50	16.25	13.75	91.25	88.75	40	30	-5	65
8	35	10	0	45	12.5	12.5	82.5	82.5	50	40	0	90
9	35	10	0	45	13.75	13.75	83.75	83.75	50	40	0	90
10	30	10	0	40	15	15	80	80	50	40	0	90
11	15	5	0	20	12.5	13.75	57.5	58.75	40	40	0	80
12	35	15	0	50	15	15	90	90	50	50	0	100
13	15	5	0	20	15	15	60	60	10	30	0	40
14	30	10	0	40	13.75	15	78.75	80	50	50	0	100
15	30	15	0	45	13.75	13.75	83.75	83.75	50	50	0	100
16	35	15	0	50	15	12.5	90	87.5	50	50	0	100
17	35	10	0	45	12.5	12.5	82.5	82.5	50	50	0	100
18	35	10	0	45	15	15	85	85	50	30	0	80
19	35	15	0	50	15	15	80	80	40	30	0	70
20	30	0	0	30	15	15	70	70	10	0	-20	0
21	35	10	0	45	15	15	85	85	50	40	0	90
22	30	10	0	40	15	13.75	80	78.75	30	30	-5	55
23	15	10	-5	20	15	15	60	60	40	30	0	70
24	30	10	0	40	16.25	15	81.25	80	40	50	0	90
25	30	15	0	45	11.25	12.5	81.25	82.5	40	50	0	90
26	30	10	0	40	12.5	13.75	77.5	78.75	50	40	0	90
27	35	15	0	50	12.5	13.75	87.5	88.75	50	50	0	100
28	30	10	0	40	15	11.25	80	76.25	50	40	0	90
29	30	10	0	40	11.25	12.5	76.25	77.5	40	50	0	90
30	35	15	0	50	11.25	12.5	86.25	87.5	40	30	0	70

None of the patients had any malalignment, component malposition, loosening, or patellar maltracking; hence, the score for malalignment was ‘0’ for all the patients. The AP and mediolateral stability was within 0-5 mm for all the knees; hence, the score for stability was 25 for all the patients. None of the patients had any flexion deformity or extensor lag exceeding 5° and 4°, respectively, and hence the scoring for these factors was ‘0.’ Correlation between the pain score and the functional score and that between range of motion and functional score was not found to be statistically significant in either of the groups at 95% confidence interval [Table 6].

**Table 6:** Correlation between pain score, range of motion and functional score

Correlation between	Spearman‘s correlation coefficient (r)		*P* value (95% confidence interval)	
	Unilateral group	Bilateral group	Unilateral group	Bilateral group
Pain score and	0.338	0.326	0.068	0.079
Functional score				
Range of notion score	0.236	Left: -0.114	0.209	Left: 0.547
and Functional		Right: -0.196		Right: 0.299
score				

## DISCUSSION

A persistent limp is one of the most frustrating symptoms after total hip arthroplasty. There are many causes of limp; however, leg length discrepancy is one of the common reason for litigation after an otherwise successful total hip arthroplasty.^15^–^17^ Limb length discrepancy is discussed in depth in the literature with respect to hip replacement but there are few studies that have examined limb length dicrepency following total knee arthroplasty.^3^,^18^,^19^ Hence, we conducted this analysis Although the sample size is small but the power of study with the given sample size is 86%, making the results statistically acceptable.

Bhave *et al*.^3^ showed that the operated leg gained length compared to the contralateral unoperated leg due to correction of the varus deformity. This discrepancy resulted in a flexed knee posture and a resultant knee flexion contracture. In our study also the unilateral group had a limb length discrepancy (i.e. the operated limb gained length) ranging from 0 centimeter to 3 cm. In the unilateral group, 83.33% of the patients had limb length discrepancy. In contrast, in the bilateral group only 46.66% had a limb length discrepancy, which ranged from 0 to 2 cm. This shows that limb length discrepancy is more common among those operated for unilateral total knee arthroplasty than in those operated for bilateral total knee arthroplasty. In contrast to the findings of Bhave *et al*, none of the patients included in this study had any flexion contracture.

In the unilateral group 83.33% patients had some limb length discrepancy. The functional score in this group showed a statistically significant negative correlation with limb length discrepancy. This finding is in keeping with that of Gurney *et al*.,^20^ who showed that elderly people have difficulty walking even with a limb length discrepancy as small as 2 cm. The limb length discrepancy induces quadriceps fatigue in the longer leg. Mahar *et al*.,^21^ also showed that limb length discrepancy of as little as 1 centimeter was biomechanically significant enough to produce eccentric forces at the joints of the lower limb and spine. These forces continued to increase in proportion to the magnitude of the discrepancy.

In the bilateral group, although 46.66% patients had some limb length discrepancy, there was no statistically significant correlation between the functional scores and the limb length discrepancy. This shows that a limb length discrepancy in a patient operated for unilateral total knee arthroplasty has a statistically significant effect on the functional outcome of the patient.

None of the patients operated for bilateral total knee arthroplasty perceived any limb length discrepancy. Of the patients operated for unilateral total knee arthroplasty, those who perceived limb length discrepancy had a discrepancy of 2 cm or more. Patients having limb length discrepancy less than 2 cm (even in the unilateral group) did not perceive any limb length discrepancy.

Patients with unilateral total knee arthroplasty had a significant increase in functional scores after being operated for the other knee. The limb length discrepancy present after the unilateral surgery got corrected after the second surgery. The two patients who perceived limb length discrepancy after unilateral total knee arthroplasty did not do so after the second surgery.

A number of factors may be responsible for the limb length discrepancy, including correction of the varus alignment after surgery, the amount of preoperative flexion deformity, and the postoperative flexion deformity. Investigation for these factors was not the aim of this study and hence we have not considered these variables. None of the patients in the study had any malalignment, component malposition, patellar maltracking, or instability. Thus, these factors did not act as confounders in this study.

The mean limb length discrepancy after total hip arthroplasty varies from 1 to 15.9 mm.^22^,^23^ Limb length discrepancy after total hip arthroplasty is perceived by 32-43%^18^,^19^ of patients. Also limb length discrepancy is one of the factors influencing the difference in postoperative Oxford hip score.^19^ In comparison, in this study on limb length discrepancy after total knee arthroplasty, the limb length discrepancy varied from 0 to 3 cm. Limb length discrepancy was perceived by 26.7% of patients operated for unilateral total knee arthroplasty. All these patients had limb length discrepancy of 2 cm or more. Also, the limb length discrepancy had a negative correlation with the postoperative functional knee score. Thus, it appears that after total knee arthroplasty for patients with bilateral varus knee osteoarthritis, limb length discrepancy affects functional outcome in a similar way as after total hip arthroplasty.

## CONCLUSION

Limb length discrepancy is more common after unilateral than after bilateral total knee arthroplasty (83.33% vs 46.66%). Limb length discrepancy of 2 cm or more is perceived by patients operated for unilateral total knee arthroplasty. Limb length discrepancy of 2 cm is not perceived by patients operated for bilateral total knee arthroplasty. Limb length discrepancy after unilateral total knee arthroplasty for bilateral varus osteoarthritis significantly affects the functional outcome, but the same is not true for patients operated for bilateral total knee arthroplasty. The functional outcome of patients of bilateral knee osteoarthritis with varus deformity operated for unilateral total knee arthroplasty improves significantly after being operated for the other side.
